# The Book World of Medicine and Science

**Published:** 1894-12-08

**Authors:** 


### The Book World of Medicine and Science.

### ARMY MEDICAL ORGANISATION.

The United Service Magazine for December contains a very sensible article upon Army Medical Organisation, evidently written by some one fully conversant with the difficulties and aspirations of the Military Medical Service. After reviewing briefly the various causes of the evolution of the present system, the writer unhesitatingly and adversely criticises the administration of the Medical Department during 1882-1889, during which period errors of administration and want of foresight on the part of the chief of the department have resulted in the present unsettled condition of what should be a powerful and homogeneous service.

The writer alludes to the present regulations for admission to the service with more or less approval, but we think might have strengthened his criticism had he pointed out the great need of getting men to join the army at earlier ages than they now do, and the importance of the authorities insisting upon all candidates for commissions having served at least six months as house surgeon or house physician in some large hospital, coupled with a stringent test of general education and culture. The need of this latter is particularly emphasized in the army, where medical officers are frequently thrown in intimate contact with the ordinary officer, who now receives more than average education.

Any proposed scheme of short service for the Medical Department is rightly shown to be impracticable; but we think much improvement might result if the present power in the hands of the authorities were more generally exercised for getting rid of men who after a few years' service have clearly shown themselves to be unfitted for military life.

The question of rank has been judiciously discussed, and the writer shows that with the present compound titles, the purely military status of the medical officer is sufficiently defined; excepting perhaps a plea for the modification of the present cumbrous title of brigade-surgeon-lieutenant-colonel, this question does not need reopening. The writer advocates, very truly, the combination of the station hospital with the allocating of medical officers to regiments. This appears to be the only true solution of the present want of touch between fighting units and the largest department in the army. This system now exists in the Guards' Brigade, and there should be no real difficulty in extending it to the army at large. It is simply a question of cost.

The following sentence is so full of common sense, and yet at the same time touches upon a vital point in the efficiency of the service, that it bears quoting verbatim; "The first duty of the head of any department, and of none more than the medical, is to make himself acqainted with the special merits of his senior officers. One medical officer may be a good physician, another a good surgeon, a third a good sanitarian, and a fourth a good organiser. While it is impossible to find all these points of excellence in one and the same person, there ought to be no difficulty for a department to be so informed that it would be able to employ its officers to the best advantage. He is the best administrator, the best leader of men, who knows how to employ those officers who serve under him. Except occasionally for the accident of field service, no medical officer has been promoted. Nothing has been done to encourage professional merit or devotion to duty." This hits the right nail on the head, and more or less puts the matter into a nutshell. If medical officers could once realise that, as good doctors and scientific men, they had a chance of material advancement, coupled with a practical recognition of the fact that their duties are those of the staff of the army, more especially of the Army Service Corps, a bright and brilliant future would be in store for a service which, notwithstanding its own errors in the past, and in the face of much military opposition, has hitherto served the country both loyally and well.

